# P-342. Outcomes of Human Metapneumovirus in Nursing Home Residents: A Matched Analysis

**DOI:** 10.1093/ofid/ofae631.544

**Published:** 2025-01-29

**Authors:** Nidhi Bhaskar, Yasin Abul, Kevin McConeghy, Frank Devone, Christopher Halladay, Stefan Gravenstein, James L Rudolph

**Affiliations:** Brown University, Providence, Rhode Island; Brown University, Providence, Rhode Island; COIN-LTSS, Providence Veterans Affairs Medical Center, Providence, Rhode Island; PROVIDENCE VAMC, Providnece, Rhode Island; Providence VAMC, Providence, Rhode Island; Brown University, Providence, Rhode Island; PROVIDENCE VAMC, Providnece, Rhode Island

## Abstract

**Background:**

Identified in 2001, human Metapneumovirus (hMPV) is a Paramyxoviridae with bimodal age distribution - affecting children and older people. In older people, clinically severe hMPV may be associated with respiratory symptoms resulting in hospitalization and/or death. The purpose of this study was to compare hospitalization and death in older nursing home residents with hMPV or influenza.

**Methods:**

Using the electronic medical records of VA Community Living Centers (CLC) during the epidemiology years of Sept 1, 2017 to August 31, 2023, we identified CLC residents who tested positive for hMPV or influenza. We collected information on demographics and comorbidities, as well as clinical testing. We developed a propensity matched cohort of hMPV and influenza positive CLC residents and used proportional hazards models to determine the hazard ratio (HR) for hospitalization, death or either during the 90 days after positive test among hMPV- and influenza-positive residents.

**Results:**

During the study period, we identified 135 hMPV infections and 1170 influenza. Most hMPV included influenza testing and there were no co-infections. Both populations were older (hMPV 76.1 (10.6) vs. influenza 74.2 (10.9) years, SMD=0.18) and predominantly male (hMPV 96.7% vs. influenza 96.3%, SMD=0.02). The hMPV cohort had more pulmonary disease (42.2% vs 30.9%, SMD=0.25) and dementia (55.6% vs. 42.3%, SMD=0.26) compared to influenza cohort. The propensity matching identified 130 hMPV and 601 influenza residents that were well matched. proportional hazard model on the propensity matched cohort found equivocal hazard for hospitalization (adjusted HR 0.91 (0.55, 1.50)), death (adjusted HR 1.03 (0.61, 1.75)), and the combined outcome (adjusted HR 0.92 (0.61, 1.33)).

**Conclusion:**

When hMPV develops in nursing home residents, the outcomes of hospitalization and death are comparable to influenza. Increased awareness of hMPV and appropriate point of care testing are needed to accurately detect hMPV and implement infection control measures. Additional studies are needed to better understand epidemiology, prevention, and treatment of hMPV in especially high-risk nursing home residents.

Funded by Icosavax, a member of the AstraZeneca Group

**Disclosures:**

**Yasin Abul, MD**, Moderna: Grant/Research Support|Moderna, Abt, CDC: Grant/Research Support **Kevin McConeghy, Pharm.D.**, Genentech: Grant/Research Support|GlaxoSmithKline: Grant/Research Support|Moderna: Grant/Research Support|Sanofi-Pasteur: Grant/Research Support|Seqirus: Grant/Research Support **Stefan Gravenstein, MD, MPH**, CDC: Advisor/Consultant|CDC: Grant/Research Support|Genentech: Advisor/Consultant|Genentech: Grant/Research Support|Genentech: Honoraria|GlaxoSmithKline: Advisor/Consultant|GlaxoSmithKline: Grant/Research Support|GlaxoSmithKline: Honoraria|Janssen: Advisor/Consultant|Janssen: Grant/Research Support|Janssen: Honoraria|Moderna: Advisor/Consultant|Moderna: Grant/Research Support|Moderna: Honoraria|NIH: Grant/Research Support|Pfizer: Advisor/Consultant|Pfizer: Grant/Research Support|Pfizer: Honoraria|Sanofi: Advisor/Consultant|Sanofi: Grant/Research Support|Sanofi: Honoraria|Seqirus: Advisor/Consultant **James L. Rudolph, MD**, Icosavax / AstraZeneca: Grant/Research Support

